# The Q-Pass Index: A Multifactorial IMUs-Based Tool to Assess Passing Skills in Basketball

**DOI:** 10.3390/s21134601

**Published:** 2021-07-05

**Authors:** Arturo Quílez-Maimón, Francisco Javier Rojas-Ruiz, Gabriel Delgado-García, Javier Courel-Ibáñez

**Affiliations:** 1 Department of Physical Education and Sport, Faculty of Sport Sciences, University of Granada, 18071 Granada, Spain; arturoquilez@ugr.es (A.Q.-M.); fjrojas@ugr.es (F.J.R.-R.); gabrieldg@ugr.es (G.D.-G.); 2 Department of Physical Activity and Sport, Faculty of Sport Sciences, University of Murcia, 30720 Murcia, Spain

**Keywords:** biomechanics, motor control, 3D analysis, team sport, gyroscope, accelerometer

## Abstract

Despite being a key sport-specific characteristic in performance, there is no practical tool to assess the quality of the pass in basketball. The aim of this study is to develop a tool (the quality-pass index or Q-Pass) able to deliver a quantitative, practical measure of passing skills quality based on a combination of accuracy, execution time and pass pattern variability. Temporal, kinematics and performance parameters were analysed in five different types of passes (chest, bounce, crossover, between-the-leg and behind-the-back) using a field-based test, video cameras and body-worn inertial sensors (IMUs). Data from pass accuracy, time and angular velocity were collected and processed in a custom-built excel spreadsheet. The Q-pass index (0–100 score) resulted from the sum of the three factors. Data were collected from 16 young basketball players (age: 16 ± 2 years) with high (experienced) and low (novice) level of expertise. Reliability analyses found the Q-pass index as a reliable tool in both novice (CV from 4.3 to 9.3%) and experienced players (CV from 2.8 to 10.2%). Besides, important differences in the Q-pass index were found between players’ level (*p* < 0.05), with the experienced showing better scores in all passing situations: behind-the-back (ES = 1.91), bounce (ES = 0.82), between-the-legs (ES = 1.11), crossover (ES = 0.58) and chest (ES = 0.94). According to these findings, the Q-pass index was sensitive enough to identify the differences in passing skills between young players with different levels of expertise, providing a numbering score for each pass executed.

## 1. Introduction

Sport scientists and coaches are provided with low-cost, reliable and wearable technology to monitor athletes’ performance and assist them in training activities, such as inertial measurement units (IMUs) [1,2]. In recent years, researchers, information and practitioners have gained the possibility of using smart phone devices since most of them have a built-in gyroscope and accelerometer, which provides detailed information to researchers that might be used as the basis of the development of ecological dynamic approaches [3,4]. These IMUs are small and portable, allowing the players to move freely and naturally on the court while recording motion in terms of linear acceleration and angular velocity [5]. Due to its versatility and accuracy, 3D motion analysis and IMUs have been widely used in the evaluation of technical skills in sports like handball or baseball [6,7,8], which supports the use of objective measures in learning contexts, particularly in motor skills learning. However, there is a lack of studies including inertial sensors for technical assessment in basketball. This seems to be particularly useful in fundamental overarm movement skills [8], like passing a ball to a team-mate.

Passing skill is a key variable in performance and one of the biggest factors in selection and talent development process [9]. During the offence, players are required to pass the ball and cooperate to create optimal shooting options to increase effectiveness. Passes must be accurate and quick to reach the target (a free teammate) and avoid the opponent to intercept the ball. Previous studies examining professional basketball players concluded that the two-handed chest pass was the most common and easiest [10]. Besides, other authors highlighted the importance of the ability to perform quick passes in modern basketball, thus improving the one-hand passing skills and passing right after bouncing or dribbling are primary training goals [11]. Because team sports like basketball are constantly forcing the players to adjust their motion depending on the game conditions, the assessment of passing skills and performance should be made under uncertain and variable conditions to obtain information on players’ responses to competitive scenarios [12]. Under these collective scenarios in where players are interacting, variability in a real situation context is a sign of expertise as the performer demonstrate their ability to adapt to the performance context [13,14]. In turn, the absence of competition (e.g., performing 1 × 0 situations with no opponents), highly skill players are characterised by stable movement patterns that are consistent over time, resistant to perturbations and reproducible in that a similar movement pattern may recur under different task and environmental constraints. Hence, little or negligible variability in repeated executions would mean that the player has acquired a good technical gesture by warrantying a high and reproducible performance, while a significant variability is detrimental or indicates a weakness in the performance [15,16]. In tasks of a closed nature, it is easier to identify the optimal movement technique, taking into account that factors exogenous to the athlete (or environmental factors) play a less important role in the modelling equation [17]. On the other hand, numerous authors have used an expert model to correct sports technique [18,19,20,21].

Biomechanical factors are a limiting factor for performance. Along these lines, some authors have developed biomechanical evaluation tests in open-nature sports [22,23]. A very few studies have investigated the basketball passing skills in terms of biomechanical parameters [12]. Only one previous study has explored the influence of both reaction and execution time during two different basketball passing tests that implied tasks under laboratory conditions [24]. In a more practical sense, other authors explored various passing techniques among 150 games in top-level leagues (Italian League, NCAA and NBA), concluding that the two-handed chest was the most common and easiest pass type, while the one-handed was the second most common but the least effective [10]. Nonetheless, it has been argued that improvements in low time-consuming actions such as the one-hand passing skill stands as a primary training goal in modern basketball [11]. It is remarkable that no previous study has explored the movement variability in basketball passing [25].

Movement quality is described as the way in which human movements are executed with respect to the dimensions of time and space [26]. It has been evaluated in different ways, such as: (I) Subjective ability to focus on perception and to perceive the whole body, postural self-control, ability to relax mind and body [27]; (II) assessed by systematic observation of motor activity, based on knowledge of how the relevant tasks are performed by subjects with normal and disturbed motor activity [28]; (III) using biometric parameters associated with movement such as muscle stiffness in movement [29], kinematic parameters [30,31] or spatial movement characteristics [32]. In that way some authors have designed a specific tool for evaluating the quality of movement [33,34,35]. In basketball, the evaluation tools used to investigate the passing quality of the pass mainly consisted of performing field-based passing tests with results exclusively based on whether the pass is successful or not, in which the greater number of scores (i.e., hits on a target) represent a better skill [36,37,38]. Field-based tests are widely adopted as they require low-cost equipment and are easily accessible for practitioners and researchers. Nonetheless, the assessment of passing skills based only on success rates can be insufficient to determine the quality of the pass or to distinguish between high- and low-skill players [16]. Therefore, the development of alternative tools to accurately measure the quality of the pass in basketball is desirable.

Owing to technology development, nowadays passing skills parameters such as movement time and motor variability can be accurately measured with wearable inertial sensors (IMUs) while performing training tasks or field-tests. On these grounds, what is now required is to provide coaches with practical tools to collect and interpret the IMUs data to be used in the real world for taking better decisions during the training process and assist in player talent development and identification. Furthermore, inertial sensors have been used in previous work to assess the quality of movement [39].

Therefore, the aims of this study are: (i) To quantify a set of temporal, kinematics and performance parameters in five different types of passes, using video cameras and body-worn IMU sensors; (ii) to develop a quantitative and more practical measure of quality (the Quality Pass or Q-Pass index), based on references, metrics and algorithms related to three key factors: success rates in reaching pass target, pass execution time and pass pattern execution; and (iii) to verify whether the Q-Pass and its related factors are effective in identifying differences in basketball passing skills among young players with different level of expertise.

## 2. Materials and Methods

### 2.1. Experimental Design

**Figure 1:**
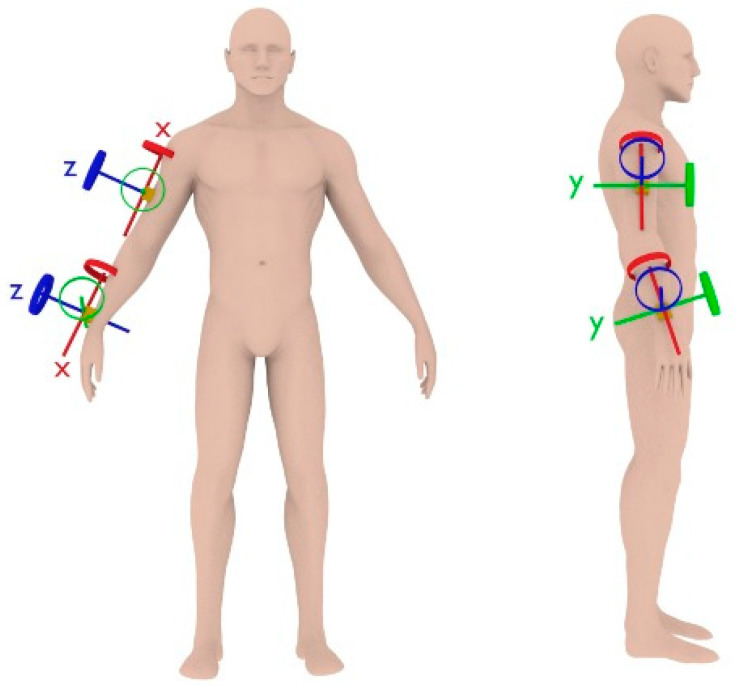
IMUs location and relevant coordinate systems in upright standing can be appreciated: Z-axis, anteroposterior; Y-axis, mediolateral; X-axis, vertically aligned with the direction of the gravitational field vector.

This is a cross-sectional study conducted on two separate days (one per experimental group), during stable weather conditions. Prior to the evaluations, participants completed a familiarisation of the full protocol during the previous week. Initially, five IMU sensors were placed on both players’ arms and trunk [40]. Two IMU sensors were placed on players’ dominant arm. The rationale of this choice was based on previous similar studies [8] and according to our own preliminary results using five sensors (dominant arm and forearm, non-dominant arm and forearm and centre of mass) during familiarisation. Two video recording cameras (CASIO EX-ZR800) were employed to record players’ performance at 210 Hz. Both cameras were synchronised with IMU signals using a Wi-Fi signal. Participants completed a basketball passing test using an official ball-size (76 cm, 0.490 kg) in their regular training environment. Data obtained from the IMU and the video cameras were used to calculate the quality pass index (Q-Pass). Participants anthropometric characteristics were measured using standard procedures to accurately place two IMU sensors (I2M NexGen Ergonomics Inc, Canada, 2017; fs = 128 Hz, dimension: 43.7 mm × 39.7 mm × 13.7 mm, weight: 0.250 kg): one in the wrist and one in the medial zone of the humerus (Figure 1). Sensors were placed on the players’ dominant arm using elastic belts to keep the devices fixed. After placing the devices, players were asked to perform basketball actions such as dribble, pass and shoot as a part of the familiarisation to ensure comfortability. Each IMU comprised a 3-axis accelerometer, a 3-axis gyroscope and a 3-axis magnetometer. The scalar components of axes (*x, y, z*) out of the vectors information from the two gyroscopes signals were used in this study following previous recommendations [8,41]. Data were exported to a laptop and processed using the software provided by the manufacturer and an Excel sheet.

### 2.2. Participants

Sixteen young male basketball players (age: 16 ± 1.9 years, height: 173 ± 12 cm; body mass: 70.4 ± 10.2 kg) volunteered to participate in this study. Players were classified according to their level of expertise: A-level (advanced players with more than seven years competing at federated level; *n* = 8) and B-level (novice players with four or less years competing at regional level; *n* = 8). All the participants were competing at federated level (A-level players following 6 h of training and two matches at national level per week and B-level players following 4.5 h of training and one match at regional level per week). Because players specific positions are very likely to have an impact on the different results (e.g., a guard is trained since the beginning to have a more complete and powerful control of ball bouncing, as compared to a centre, and bouncing is a necessary previous step prior to start the pass), each group included players from different specific positions: three-point guards, three forwards and two centres. All players, their parents and the teams’ supervisors were informed of the research protocol, requirements, benefits and risks, and their written consent was also obtained. The local Institutional Research Ethics Committee approved this study, and it conformed to the Declaration of Helsinki.

### 2.3. Testing Procedures

The IMUs location in the dominant hand and relevant coordinate systems in upright standing (Z-axis, anteroposterior; Y-axis, mediolateral; X-axis, vertically aligned with the direction of the gravitational field vector) is shown in Figure 1.

**Figure 2:**
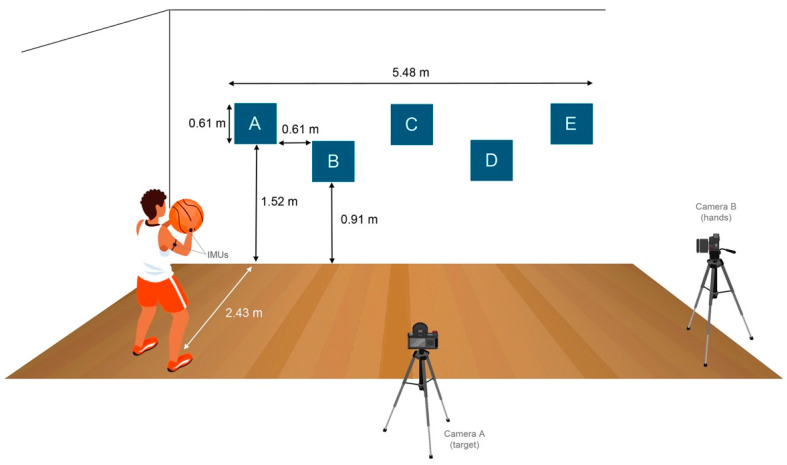
Schematic representation of the passing test, showing main elements involved: targets A, B, C, D, E, participant wearing 2 IMUs in his dominant arm, camera A facing the targets to record hits accuracies, camera B recording to determine movement time.

Participants performed a variant of the AAHPERD test, originally designed by the American Alliance for Health, Physical Education, Recreation and Dance [38,42]. This test has found to be reliable in a test-retest condition (Pearson correlation coefficients, *r* = 0.84 o 0.97) [38,42]. The test started with the participant wearing the IMUs, adopting the triple-threat position (which allows a player to dribble the ball, to pass the ball, or shoot the ball) holding the ball, standing behind a line 2.43 m from the wall, and facing five 0.61 m × 0.61 m targets located on the wall at different heights and separated 0.61 m from each other (Figure 2). Besides, two video cameras were recording and synchronised with the IMU signals: Camera A was in the front, focusing on the targets, and Camera B was in the flanked, focusing on the player’s hands.

At the signal, participants executed five consecutive passes against the first target attempting to hit the 0.61 m × 0.61 m target, receiving back the ball after it hits the wall, and then moved to the next target to repeat a new series of five passes, until completing the five stages. Every participant repeated the test five times, with 5-min rest in between, varying the passing situation: (1) straight two handed (chest), (2) one-handed pass after bouncing with dominant hand (bounce), (3) one-handed pass after changing direction from the non-dominant hand to the dominant hand by crossover (crossover), (4) one-handed pass after changing direction from the non-dominant hand to the dominant hand by bouncing through the legs (between-the-leg), and (5) one-handed pass after changing direction from the non-dominant hand to the dominant hand by bouncing behind the back (behind-the-back).

**Figure 3:**
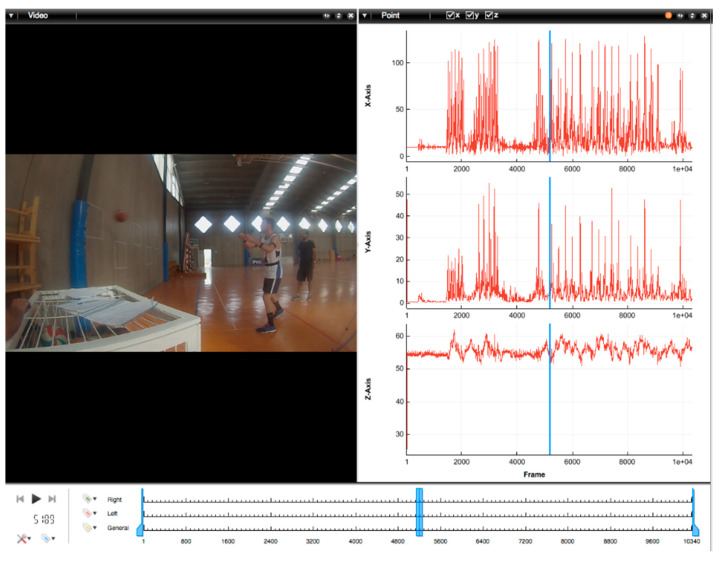
Mokka: Motion kinetic and kinematic analyser (v0.6.2), synchronised with the IMU signals to collect data from the success rates in hitting the targets (accuracy) and passing execution time.

Videos were analysed with Kinovea video-analysis software (v.0.8.15) and Mokka Motion kinetic and kinematic analyser (v0.6.2) (Figure 3) toolkits to collected data from the success rates in hitting the targets (accuracy) and passing execution time. Pass execution started when the participant just received the ball in his hand to initiate the sequence of movements specific to each pass type, and finished when the player releases the ball towards its target destination. Initial and final time stamps were precisely determined based on static images selected out of the recorded videos. Angular velocities were exported and processed using a custom-built Excel spreadsheet (Supplementary Materials).

### 2.4. Q-Pass Index Algorithms

A free access, custom-built excel spreadsheet is available in Supplementary Material. A quantitative assessment method has been designed based on the assignment of values (natural numbers) of penalty considering the three factors of, factor 1 (f_1_) achievement/success rate in reaching the final destination (the higher the hit accuracy inside the target area the lower the penalty), factor 2 (f_2_) the timing of pass execution (in general terms the lower the time spent, the lower the penalty), and factor 3 (f_3_) the pattern of technical execution (the lower the pattern variability the lower the penalty).

#### 2.4.1. Factor 1 (f_1_), Accuracy: Success Rate in Reaching the Pass Target

To assign a quantitative evaluation associated with this factor, each executed pass Ex_j_, of a given pass type PT_i_, performed by any given player Py_k_, {Py_k_ PT_i_ Ex_j_} is assigned a number of one, two or three digits (in general terms) out of five (5) potential values (0, 25, 50, 75, 100). This number is related to the percentage of the surface of the ball that hits the square area marked on the wall as a correct target area, according to the definition of AAHPERD-1984. The lines that draw the boundaries of that area are not considered part of the correct destination area. Static images out of the videos recorded by the cameras were selected at appropriate moments to be able to assign precise penalty values. Values lie between 0 (100% perfect hit within target area without touching any boundary line) and 100 (the ball hits completely outside the boundary lines that define the edges of the target domain’s target domain). The other three possible values are 25, when most of the estimated area impacts the destination area; 50, when close to 50% is within the destination area and around 50% is outside said destination area (including boundary lines); and 75, when most of the estimated surface hits outside the boundary lines.

#### 2.4.2. Factor 2 (f_2_), Time Motion: Pass Execution Time

To assign a quantitative evaluation associated with this factor, for any executed pass {Py_k_ PT_i_ Ex_j_}, a penalty number is assigned, that number being directly related to the absolute value expression [Ex_j_-Ex_Tr_], in which Ex_tj_ denotes the Ex_j_ pass execution time and Ex_Tr_ denotes a pass reference execution time. To set such comparison reference Ex_Tr_ for f_2_, the passes executed by the group of A-level players for each pass type were analysed and then the pass duration average value of the quickest pass type was used to define Ex_Tr_. Pass execution starts when the participant just receives the ball in his hand to initiate the sequence of movements specific to each pass type, and finishes when the player releases the ball towards its target destination. Initial and final time stamps are precisely determined based on static images selected out of the recorded videos. Pass execution elapsed time was calculated together with the number of samples involved, n (elapsed time = n × (1/128) s). Ex_Tr_, being the comparison reference, has a value of 0 in terms of penalty regarding the second factor f_2_. The penalty numbers obtained out of the expression [Ex_tj_-Ex_Tr_], should always be rounded to the nearest natural number. Once all the evaluations have been completed with respect to f_2_, all obtained penalty numbers are normalised to be in the range between 0 and 100 by applying a proportionality factor that assigns to the maximum penalty number the value of 100, weighting the rest of the obtained numbers accordingly. Penalties are weighted proportionally specific to this group of participants, but this particular assessment can be replicated on another cohort.

#### 2.4.3. Factor 3 (f_3_), Variability: Pattern of Technical Execution

This factor f_3_ aims to measure the variability between technical executions of successive passes performed in sequence and belonging to the same type. A low variability (ideally a variability equal to zero) directly associates with a good control by the player with respect to that pass type. In order to determine the evolution of the angular velocity during the execution of a given pass, the two vectors of the gyroscope of the dominant arm were first added vectorially in the form |gyr1 + gyr2| (adding their respective cartesian components *x*_1_ + *x*_2_, *y*_1_ + *y*_2_, *z*_1_ + *z*_2_), and then the module of the resulting sum vector |gyr1 + gyr2| was obtained over time, taking samples every 1/128 s (i.e., every 78.125 ms).

(1) $$Angular\;velocity=\sqrt{{\left(x+y+z\right)}^{2}}$$

(2) $$\left|\mathrm{gyr}1+\;\mathrm{gyr}2\right|=\sqrt{{\left[\left(x_{1}+x_{2}\right)+\left(y_{1}+y_{2}\right)+\left(z_{1}+z_{2}\right)\right]}^{2}}$$

**Figure 4:**
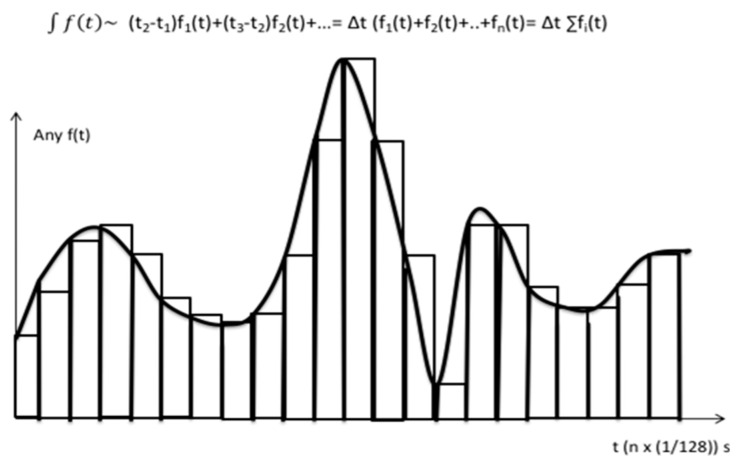
Integral approach based on elementary rectangles.

Recorded angular velocity signal was, then, low-pass filtered using a fourth-order Butterworth filter with a cut-off frequency of 25 Hz to the resulting values |gyr1 + gyr2|. If |gyr1 + gyr2| functions are the same along the time, so are the executions, thus resulting in low variabilities and high execution control. To simplify the computational load, the heavy |gyr1 + gyr2| sample to sample comparison is (approximately) replaced by the corresponding definite integral generated by the function |gyr1 + gyr2| initial and final pass execution time stamps. This integral is in turn approximated by the expression ∫f(t)~ (t2-t1) f1(t) +(t3-t2) f2(t) +… = Δt [(f1(t) + f2(t) + f3(t) +…] = Δt ∑ fi(t) see Figure 4, which in this case corresponds to Δt ∑[gyr1 + gyr2].

To set the comparison reference ExPT_ir_ for f_3_, the passes executed by the group of A-level players for each pass type PT_i_ were analysed. For each PT_i_ the mean value of all patterns of technical executions, approximated by the corresponding expression Δt ∑|gyr1 + gyr2| as mentioned before, was used to define ExPT_ir_. There are therefore, 5 ExPT_ir_ references, i = 1, ..., 5. ExPT_ir_, being the comparison reference, has a value of 0 in terms of penalty regarding the third factor f_3_ for each PT_i_. To assign a quantitative evaluation associated with this factor f_3_, for any executed pass {Py_k_ PT_i_ Ex_j_}, a penalty number is assigned for each PT_i_, that number being directly related to the absolute value expression [Δt ∑ |gyr1 + gyr2| − ExPT_ir_]. The numbers obtained should always be rounded to the nearest natural number. Once all the evaluations of each PT_i_ have been completed with respect to f_3_, all obtained penalty numbers are normalised to be in the range between 0 and 100 by applying a proportionality factor that assigns to the maximum penalty number of each PT_i_ the value of 100, weighting the rest of the obtained numbers accordingly. Penalties are weighted proportionally specific to this group of participants, but this particular assessment can be replicated on another cohort by applying same algorithms in each particular group.

#### 2.4.4. Q-Pass Index Calculation

In order to assign overall quality values, penalty values were first assigned considering the three factors. Factors f_1_, f_2_, f_3_ and its weighted combination *x*_1_f_1_ + *x*_2_f_2_ +*x*_3_f_3_ have all assigned quality values between 0 and 100, applying algorithms that are related to the percentage of the surface of the ball that hits the square area marked on the wall as a correct target area (for f_1_), the movement time or time elapsed during pass execution (for f_2_) and the evolution of the angular velocity along the pass time execution (for f_3_). The respective weights (*x*_1,_ *x*_2,_ *x*_3_) of factors (f_1,_ f_2,_ f_3_) satisfy the equation *x*_1_ + *x*_2_ + *x*_3_ = 1 and are configurable case-by-case by the coach. This allows the use of the tool towards specific objectives in each case by configuring the weighting factors accordingly (i.e., *x*_1_ = 0, *x*_2_ = 0, x_3_ = 1 would focus the evaluation on the variability of the technical execution exclusively).

### 2.5. Statistical Analyses

Preliminary analysis was done to verify the assumptions to apply further calculations. We examined z-score to identify outliers, then conducted Shapiro Wilk test for checking normal distribution and Levene’s test for homogeneity of variances. The modified mean for each factor and the Q-Pass index was calculated for each passing situation by excluding the maximum, minimum and median values. The reliability of the Q-Pass index was analysed by calculating the standard error of measurement (SEM) to determine the reliability of a single individual’s values on repeated testing (i.e., within-subject variation). SEM was calculated from the square root of the mean square error term in a repeated-measures ANOVA [43]. Coefficient of variation (CV) was computed in percentage as SEM/sample mean·100. Student’s t-test was used to identify the mean differences (95% CI M_diff_) and percentage of change in each factor (success rate, movement time and variability) and Q-Pass index scores between A-level and B-level players. The effect size was calculated by Hedge’s *g* using a pre-set spreadsheet [44] and interpreted as 0.2 small effect, 0.5 medium effect, ≥0.8 large effect and ≥1.2 extra-large effect [45]. Statistical analyses were conducted using MedCalc Statistical Software version 18.2.1 (MedCalc Software bvba, Ostend, Belgium) and IBM SPSS v. 20.0 (Armonk, NY, USA: IBM Corp.).

## 3. Results

Table 1 shows the results from the Q-Pass index reliability and mean comparisons between A-level and B-level players. A-level players obtained greater Q-Pass index in all passing situations, by order of difference: behind-the-back (21.3% greater; ES = 1.91), bounce (17.6% greater; ES = 0.82), between-the-legs (8.7% greater; ES = 1.11), crossover (7.1% greater; ES = 0.58) and chest (6.8% greater; ES = 0.94). Reliability analyses showed a high consistency among players in all passing situations (SEM between 1.9 and 7.4 points, CV between 2.8% and 10.2%).

**Figure 5:**
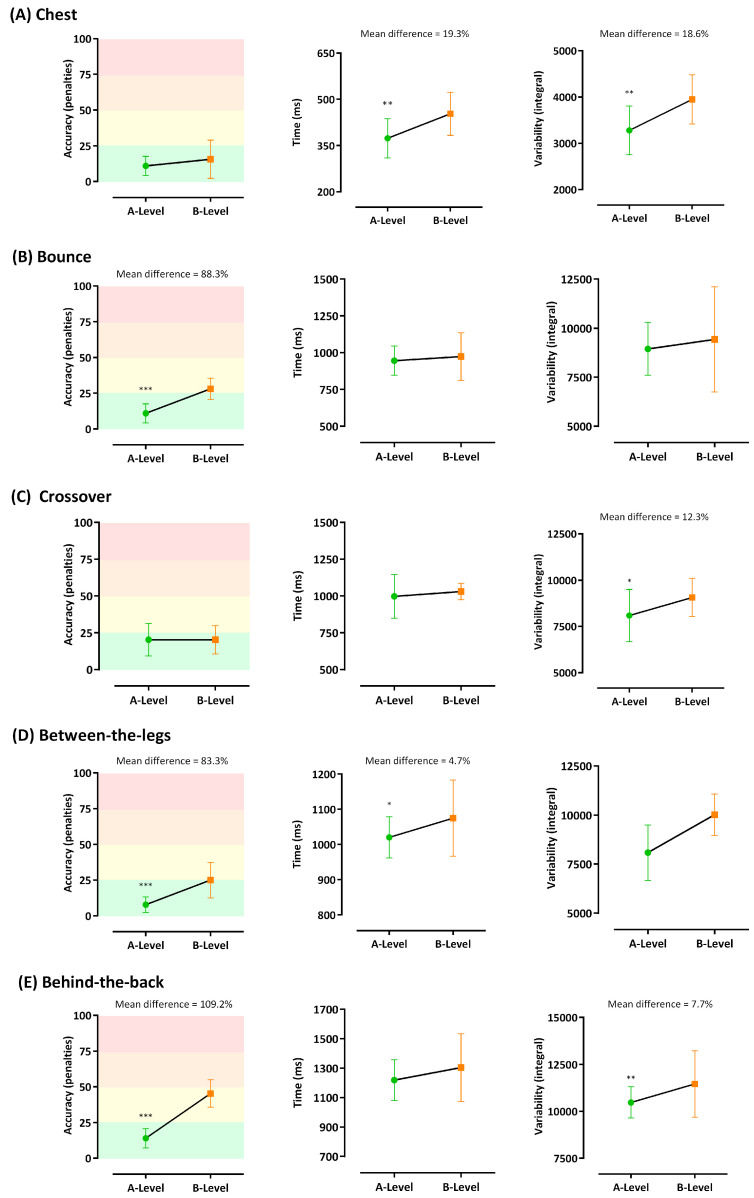
Means Means differences in passing factors (columns: accuracy, time and variability) for each type of pass (rows: (**A**) Chest, (**B**) Bounce, (**C**) Crossover, (**D**) Between-the-legs, (**E**) Behind-the-back.

Figure 5 depicts the mean differences between A- and B-level players for the separate factors related to the Q-Pass. Accuracy or success rate (Factor 1) showed extra-large effects in bounce (88.3% greater in A-Players), between-the-leg (83.3% greater in A-Players) and behind-the-back (109.2% greater in A-Players). Movement time (Factor 2) showed medium and large effects in between-the-leg (4.7% shorter in A-Players) and chest (19.3% shorter in A-Players), respectively. Variability (Factor 3) showed medium effects in crossover (12.3% lower in A-Players), and behind-the-back (7.7% lower in A-Players), but large effects in chest (18.6% lower in A-Players).

## 4. Discussion

The main findings of the current study were: (i) The combination of affordable assessment methods such as video cameras, body-worn inertial sensors and field-based test was highly effective to evaluate temporal, kinematics and performance parameters providing numerical values for each pass executed in five different types of basketball passes during formative stages; (ii) the Q-Pass index and related factors (accuracy, movement time and variability) were sensitive enough to identify differences in passing skills between young players with different level of expertise; and (iii) this novel methodology might be applicable in future studies built on game situation contexts with changing environments uncertainty which would contribute a new insight into the skill development in sport from an ecological dynamics approach. In particular, this is the first time that a practical tool for the quality evaluation of a basketball pass has been provided. The results show that the Q-Pass index and related factors are effective and feasible for the assessment of the passing skills based on quantitative motion data.

This study has training implications for team sport coaches and researchers to improve skill assessment. The combination of field tests with motion analysis systems allow to objectively quantify and identify changes in athletes’ performance by kinematic analysis within a natural competitive environment [6,7,46]. However, most of the previous studies have only included the accuracy as a specific factor of basketball passing performance, which seems to be insufficient to identify expert players [16]. Only a few studies concern about movement time in basketball passing [24] yet there are no previous studies that have included variability measurements as a specific performance parameter to assess the basketball pass quality [25]. In this sense, the Q-Pass emerges as a valid and practical tool to assess developmental changes in basketball passing skills in field conditions.

As mentioned in the methods section the Q-Pass Index can be modified giving different weight to the three factors. As in the present manuscript the age of the participants was of 16 ± 1.9 years we decided to give importance to the accuracy factor taking into account that they probably have a mature throwing pattern [47]. In prepubertal athletes, where technique could be more important than the result of the action, the precision factor could be given less weight in the equation. As in the present manuscript our findings showed that A-level players (i.e., highly skilled) obtained higher Q-Pass scores by means of greater effectiveness (higher accuracy), faster movements (less movement time) and more repetitive patterns (lower variability of technical execution) compared to the novice. These results confirm previous research indicating that A-level players (more experienced) perform better passes than B-level players [9,23]. In accuracy terms, experienced players were significantly better in bounce pass, between-the-leg and behind-the-back passes which require a greater technical complexity as it necessarily involves a prior technical action (in this case, the bounce). In addition, A-level players showed better results in movement time than B-level players, most notably in chest pass.

**Figure 6:**
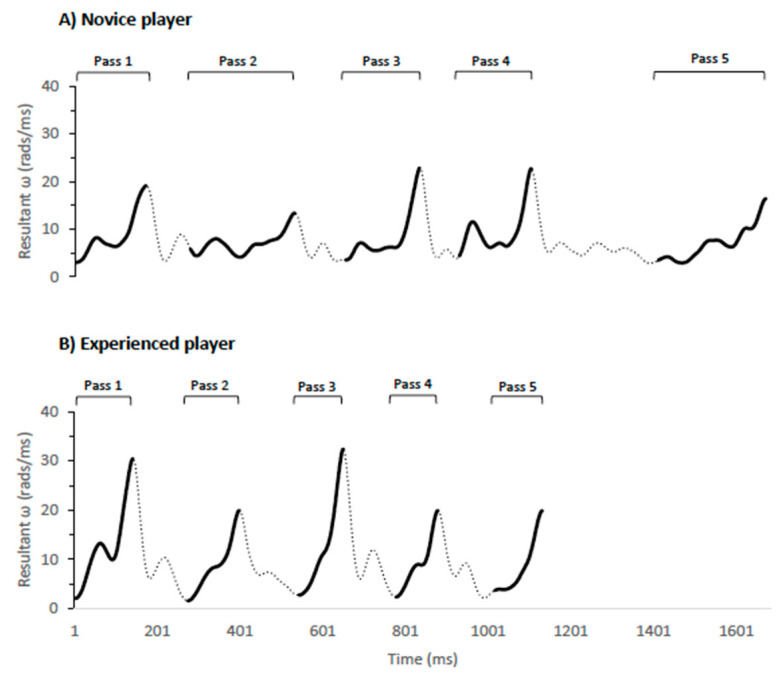
Differences in several passes performed by one experienced and one novice player.

These findings are consistent with previous studies showing that more experienced players execute faster passes which lead to a better performance [48]. Skill-specific training appears to be a learning facilitator that may reduce the movement time and increase the consistency of the technical action [24]. As an example, Figure 6 depicts the sequence of five between-the-leg passes performed by one A-level player and one B-level player. It can be seen that the experienced player achieved a greater resultant angular velocity and shorter movement time in each pass, completing the sequence in a shorter time. This greater efficiency will allow the ball reaching the target faster which makes difficult the defence anticipation and increase the success ratio [49]. The fact that more experienced players showed this better ability when passing after dribbling could be explained by the need of performing previous technical actions to overcome the defence and give an accurate and intense pass to avoid the defensive reaction and let the teammate receive under optimal conditions [3].

Finally, as could be expected, experienced players showed a little variability in all passing situations compared to the novices, particularly in chest pass, behind-the-back and crossover passes. This is in line with the previous research suggesting that low variability among technical repetitions indicates a higher performance [15,16]. Altogether, these differences observed between A-level (experienced) and B-level (novice) players evidence that the success of the basketball pass might rely on the ability of the players to dominate the one-handed pass and concatenate a previous action with the ball (i.e., bounce, crossover or dribbling). Therefore, acquiring these technical skills in formative stages is essential to better cope with task constraints as increasing complexity [50].

The current investigation is not exempt of some limitations. Because of the small sample size this data should be interpreted with caution. In addition, time motion analysis required appropriate expertise when analysing video footage [51], as the analyst must identify each movement performed, which is vulnerable to human error [52]. Furthermore, while the testing protocol tool can be easily used and reproduced in a variety of situations, the current results should be only applicable to the current sample and passing types explored. Thus, it would be convenient to conduct further studies to confirm the discriminative power of the Q-Pass index among age cohorts of players and demonstrate the feasibility of IMUs and camera-based methods in players’ development and selection process. Furthermore, because of the small sample size, we were unable to conduct separate analysis comparing the differences among players’ role. Future studies should explore larger sample sizes to confirm whereas the index is more sensitive for particular players specific positions. The evaluation of the basketball pass must also take into account the decision-making process [53], so the Q-Pass Index should be complemented with other types of tests where the ability to make decisions will be evaluated similar to what other authors have suggested [54]. The Q-Pass index could be complemented, for example, with the methodology proposed by [53] or crossing the data obtained with the Q-Pass Index with data measured in the competition situation, either by obtaining game analytics or using systematic observation techniques. In this line future studies are needed in order to replicate this methodology in situations under real conditions to assess either accuracy/success rate, variability or movement time within the game context which means real basketball game actions. Finally, it would be interesting to implement the Q-Pass index as a mobile phone app, using the inertial sensors standalone or synchronising them with another phone camera to complement it with qualitative information.

## 5. Conclusions

This study put forward a practical application that may discriminate the passing ability for the valuation of a player as a distinguishing factor between player’s skills. This system allows a players’ talent-spotting through the Q-Pass index, which seems to be a determinant tool to evaluate players’ passing skills. This method allows any interested person in this context, taking into account that gyroscopes used in this study are also included in most of the smart phones on the market, to quantify the quality of certain specific actions objectively based on three quality factors in field environment providing this method a multifactorial nature. Thus, researchers are able to determine player by player which factor (or combination of factors) is decreasing each player’s performance. Once factors have been identified, a specific practice program should be established in order to improve these factors and therefore to improve the players’ capacity of passing. Researchers are able to measure the progress made whilst comparing the pre-post results. Although this study has focused on basketball passes, the tool developed might potentially be applied to any specific technical sporting gesture. It is with this in mind that it is thought that this paper might have a relevant practical application and utility in the scientific sports community.

## Figures and Tables

**Table 1:** Q-Pass index means distribution and reliability between basketball players with different playing experience.

Q-Pass Index	M ± SD		SEM		CV	
	A-Level	B-Level	A-Level	B-Level	A-Level	B-Level
Chest	80.4 ± 4.7 **	75.1 ± 5.8 **	2.9	3.4	3.6%	4.6%
Bounce	68.3 ± 6.5 **	61.1 ± 9.8 **	1.9	4.8	2.8%	7.9%
Crossover	68.6 ± 9.9 *	63.5 ± 6.0 *	4.5	4.5	6.5%	7.4%
Between-the-leg	72.7 ± 4.5 **	66.7 ± 5.6 **	7.4	5.9	10.2%	8.9%
Behind-the-back	68.1 ± 5.5 ***	52.6 ± 9.4 ***	3.9	4.9	5.8%	9.3%

Note: Effect size (ES): * medium, ** large, *** extra-large. SEM: standard error of the measurement. CV: coefficient of variation.

## Data Availability

The data presented in this study are available on request from the corresponding author. The data are not publicly available due to privacy.

## Supplementary Materials

The following are available online at https://www.mdpi.com/article/10.3390/s21134601/s1. Excel spreadsheet for the Q-Pass Index calculation.
